# Gelfoam embolization or ^125^I seed implantation may be a more effective treatment than surgical treatment for giant benign sacral neurogenic tumors

**DOI:** 10.1186/s12957-015-0662-y

**Published:** 2015-08-15

**Authors:** Xiaojun Ma, Sun Wei, Chunxi Yang, Yingqi Hua, Jiakang Shen, Zhengdong Cai

**Affiliations:** Department of Orthopedics, Tongji University, Shanghai Tenth People’s Hospital, 301 Yanchang Rd, Shanghai, 200072 China; Department of Orthopedics, Shanghai Jiao Tong University, Shanghai First People’s Hospital, No.100 Haining road, Hongkou district, Shanghai, 200080 China

**Keywords:** Gelatin sponge particle embolization, Iodine-125 seed implantation, Neurofibroma, Sacral neurogenic tumors, Schwannoma

## Abstract

**Background:**

The goal of the present study was to assess the effects of computed tomography (CT)-guided iodine-125 (^125^I) seed implantation or gelatin sponge particle (GSP) embolization on patients with giant benign sacral neurogenic tumors.

**Methods:**

A total of 24 cases with giant sacral neurogenic tumor were performed in a retrospective study between 2000 and 2012. Nineteen cases received surgical resection, and five cases received non-surgical treatment. In surgical group, patients with type III sacral tumor had received a combined anterior-posterior approach and patients with type IV were treated with simple anterior approach. In non-surgical group, CT-guided ^125^I seed implantation or GSP embolization was applied to occlude vessels. Besides, CT scanning or magnetic resonance imaging was used to assess the size and development of tumors.

**Results:**

Two of the five patients were treated three times with GSP embolization, one had received GSP embolization combined with CT-guided ^125^I seed implantation, one case did not receive any treatment, and one patient was lost to follow-up. Patients in non-surgical group were followed up for 2–8 years.

**Conclusions:**

Our study suggested that CT-guided ^125^I seed implantation or GSP embolization treatment is very useful to slow down the development of giant benign sacral neurogenic tumors.

## Background

Sacral neurogenic tumors, which consist of schwannoma and neurofibroma, originate from sacral nerves and develop along the neural foramens into inside or outside of the sacral canal [1]. The inward growth of tumors is generally restricted because of the limited space of the sacral canal; however, the outward growth of tumors may form a giant lump anterior to the sacrum. The complete resection of the tumor is considered to be an appropriate choice for a patient with sacral neurogenic tumors [2]. Nevertheless, surgical operation is risky due to the complex anatomical characteristics of this region, excessive bleeding during surgery, and the propensity for local relapse [3].

Conservative treatments such as interventional perfusion [4], radiofrequency ablation [5], endovascular embolization techniques, and computed tomography (CT)-guided Iodine-125 (^125^I) seed implantation are alternative approaches for neurogenic tumors. Previous studies have shown that endovascular embolization techniques have been widely used in the treatment of various human disorders, such as head and neck tumors [6], skull base tumors [7], and hypervascular spinal tumors [8]. Embolization provides an effective treatment for sacral neurogenic tumors by blocking the tumor blood supply and promoting tumor shrink, deformation, and even necrosis. In addition, CT-guided ^125^I seed implantation is also applied in the treatment of lung cancer [9], prostate cancer [10], spinal metastasis, and primary paraspinal malignancies [11].

In the present study, endovascular embolization techniques or CT-guided ^125^I seed implantation was performed for the patients with giant benign sacral neurogenic tumors to assess the curative effects and outcomes of non-surgical therapy for sacral neurogenic tumors. Our study aimed to track the development of tumors and evaluate the prognosis of patients with giant benign sacral neurogenic tumors after conservative treatment with a long-period follow-up.

## Methods

### General data

In this retrospective study, a total of 24 patients (10 males and 14 females; aged 21 to 69 years) with giant sacral neurogenic tumor in Shanghai First People’s Hospital from 2000 to 2012 were recruited, including 19 cases in operation group and 5 cases in non-operation group. There were 10 cases of schwannoma and 14 cases of neurofibroma. Patients who complain of sciatica, sacrococcygeal pain, abnormal urine, perineal pain, or low back and leg pain were hospitalized, and then imageological examination showed an enlarged sacral canal. This study was approved by the ethics committee of our Shanghai First People’s Hospital.

### Treatment protocols

Patients with type III sacral neurogenic tumor received a combined anterior-posterior approach. The presacral nerves and vessels were separated, and the tumors in sacral canal were resected. Patients with type IV were treated with simple anterior approach. Briefly, the vertebral lamina was surgically excised, the sacral canal was uncovered, and then the sacral nerves were separated carefully. The postoperative complications contained poor wound healing, wound infection, sensorimotor impairment, or hemorrhagic shock.

Patients who could not tolerate the resection operation, radiation, and chemotherapy were treated with non-surgical treatment and received transcatheter arterial embolization (TAE) or CT-guided ^125^I seed (Shanghai GMS Pharmaceutical Co., Ltd, Shanghai, China) implantation. Gelatin sponge particles (GSPs) (diameter, 150–350 μm) were chosen as the materials of TAE. In order to cut off the blood supply to the entire tumor, femoral artery was punctured and GSPs were inserted into artery. The ^125^I seed implantation was performed under CT guidance for the patients with unresectable giant benign sacral neurogenic tumor. After skin disinfection, the straight needle was advanced until reaching the tumor with the patients under conscious sedation and local anesthesia. Before seed implantation, the tumor volume and ^125^I seeds dose were evaluated by a computerized treatment planning system (TPS, Prowess, version 3.02, SSGI, USA). After a post-planning evaluation, the ^125^I seeds were implanted with a safe range of particle concentration and ^125^I seeds should avoid to implant around the rectum, uterus, prostate, and nerve. The spacing between each seed pair was 0.5–1 cm. CT scanning or magnetic resonance imaging (MRI) was used to evaluate the development of tumors [12].

### Karnofsky performance status

The KPS is one of the most used methods to assess the functional status of patients with cancer [13]. The criterion of KPS index was listed as previous study [14].

## Results

### Surgical treatment

Totally, 19 patients were treated with surgical operation. Operation duration ranged from 2 to 5 h, with an average time of 3 h. Blood loss volume was 500 to 5500 mL, with an average of 2600 mL. All patients with benign neurlogical tumors underwent marginal resection, and patients with malignant neurlogical tumors underwent extended resection. Eighteen cases underwent marginal resection of distal S2 never root and kept the unilateral S1 nerve root as horizontal as possible. Another case underwent partial sacral resection (PSR) because of a wide range of S1 invaded by tumors. Negative-pressure draining was provided post-operation. Internal iliac artery ligation (IIAL) and temporary blocking of abdominal aorta were performed in four patients to control hemorrhage during operation. Four cases underwent abdominal aorta balloon block to prevent hemorrhage. Two cases had complication of cerebrospinal fluid leakage and then cured after treatment with the higher end of the bed and antibiotics. The average period of follow-up was 47 months (range, 12–98 months). Three patients with benign neurlogical tumors were found postoperative recurrence, and two patients disease-freely survived. In addition, two patients were found partially shrunken tumor and two patients underwent postoperative radiotherapy locally relapsed.

### Non-surgical treatment

The average age of patients in non-operation group was 45.2 years old (range, 32–55 years old). Three patients had received non-surgical treatment, including two cases treated three times with gelfoam embolization and one case treated with gelfoam embolization and CT-guided ^125^I seed implantation. In addition, one case with sacral schwannoma did not get any treatment due to economic reason; another one with sacral schwannoma was lost to follow-up. Characteristics of patient with non-surgical treatment were listed in Table 1. There were no tumor local recurrence and metastasis for all patients during the follow-up period. Patients treated with non-surgical treatment were followed up for 2–8 years and had normal activity with effort.

**Table 1:** Clinical features of patients received non-surgical treatment

Patient	Gender	Age	Symptoms	Category	Primary tumor size (cm)	Tumor size during treatment (cm)	Treatment protocol	Follow-up (year)	KPS
A	Male	55	Sacrococcygeal pain	Sacral neurofibroma	15 × 14 × 11	19 × 18 × 16	Gelfoam embolization + implantation of 125I seeds	5	80
B	Male	51	Low back pain and sacrococcygeal pain	Sacral neurofibroma	12 × 10 × 8	13 × 10 × 8	Gelfoam embolization (3 times)	3	90
C	Male	51	Physical examination	Sacral schwannoma	12 × 9 × 6	13 × 10 × 6	Gelfoam embolization (3 times)	2	80
D	Female	32	Sacrococcygeal pain	Sacral schwannoma	12 × 11 × 9	17 × 12 × 10	No treatment	8	80
E	Male	41	Low back and leg pain	Sacral schwannoma	8 × 7 × 7	Withdraw	Withdraw	4	80

### Typical cases

**Fig. 1:**
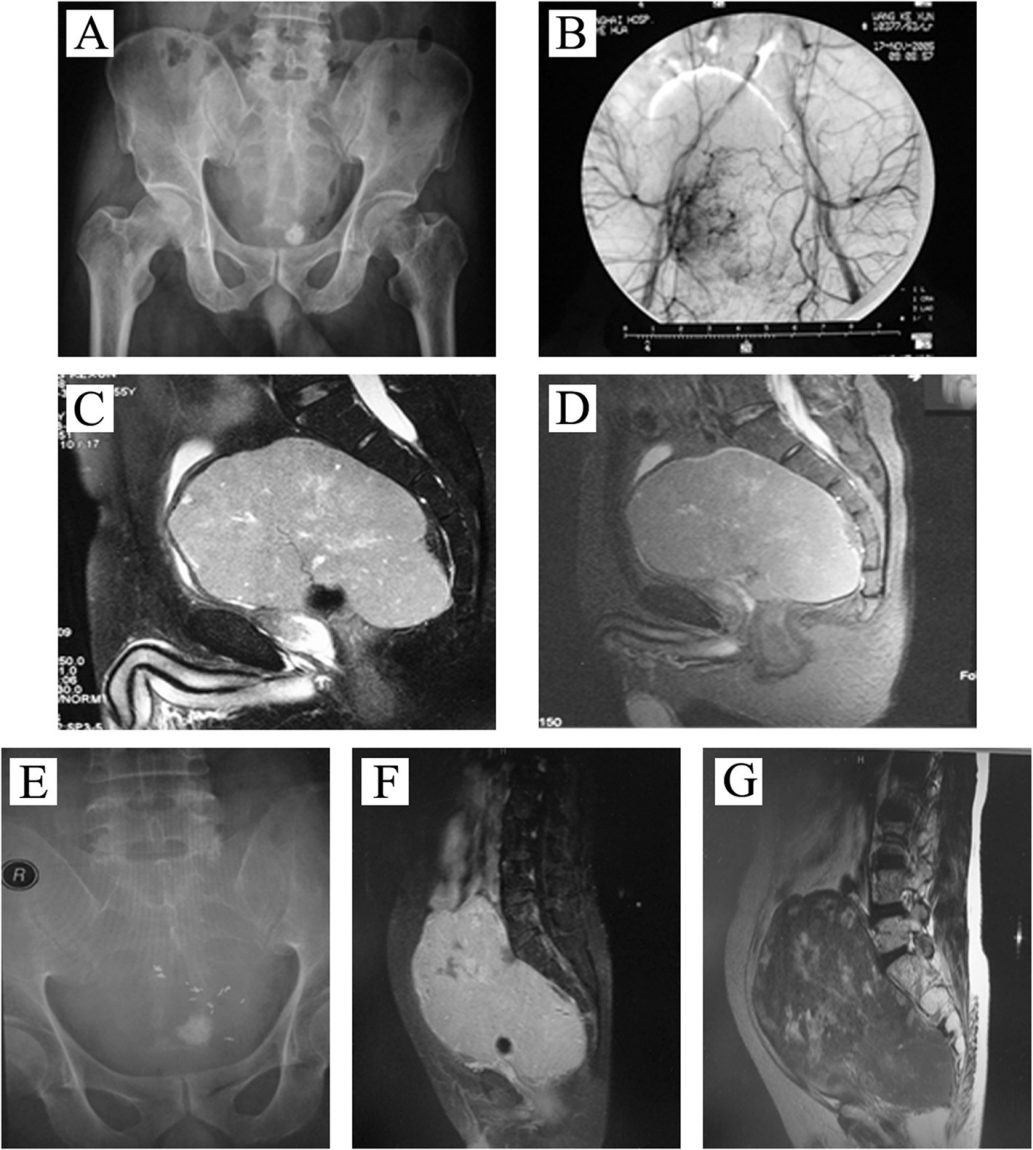
The X-ray films and computerized tomography (CT) scans of patient A before and after gelatin sponge particles (GSPs) combined with CT-guided ^125^I seed (*n* = 30) implantation treatment. **a** X-ray film of the pelvis taken in March 2005; **b** pelvic angiography showing the giant tumors before sacral in March 2005; **c**, **d** the CT scan taken in March 2005; **e** X-ray film of the pelvis taken in May 2009 after gelatin sponge particles combined with CT-guided ^125^I seed (*n* = 30) implantation; **f** the CT scan taken in October 2010; **g** the CT scan taken in September 2011

Patient A (55 years old, male), with sacrococcygeal pain and numbness in the left leg over an 8-month period, was diagnosed with sacral neurofibroma. The MRI showed that the tumors were circumscribed and exhibited lobulated shape. The primary tumor size was 15 × 14 × 11 cm. The lump was extended to the spinal channel at the S1 nerve root and superior border of coccyx. Meanwhile, the tumor implicated part of the ilium in both sides and pushed the intestinal canal in pelvic (Fig. 1a–d). The surgical operation was canceled because of his sudden cerebral infarction before surgery. So, the patient received GSP embolization and implantation of ^125^I seeds (*n* = 30) under CT guidance (Fig. 1e) in May 2009. Five years of follow-up showed that the development of tumor (19 × 18 × 16 cm) was slow-growing with a KPS score of 80 after treatment (Fig. 1f, g).

**Fig. 2:**
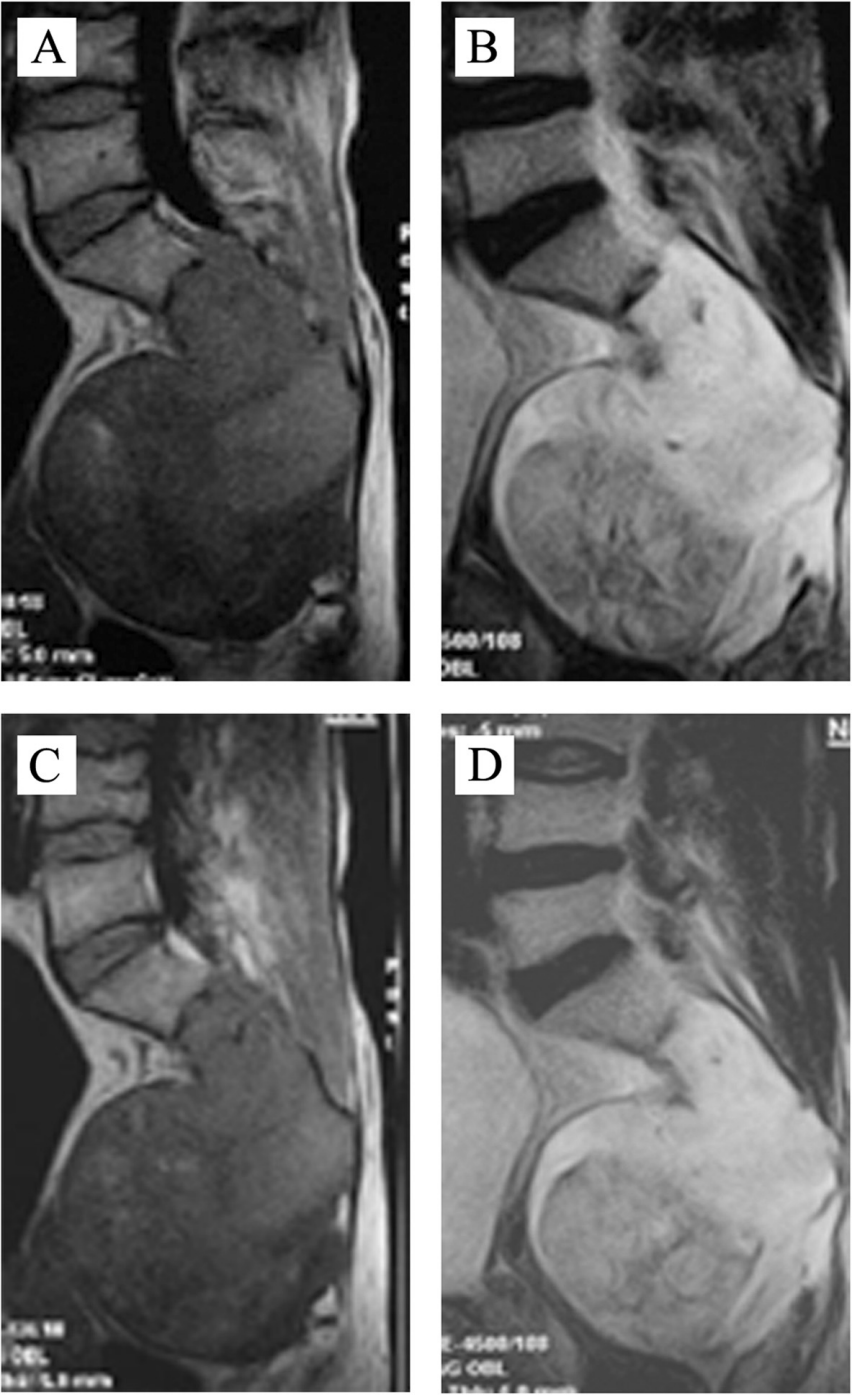
The computerized tomography (CT) scans of patient B before and after gelatin sponge particle (GSPs) implantation. Patient B received thrice of GSPs implantation in July 2011, April 2012, and December 2013, respectively. **a**, **b** The CT scans taken in February 2011; **c**, **d**: The CT scans taken in January 2014

Patient B (51 years old, male), with low back pain and sacrococcygeal pain, was diagnosed with sacral neurofibroma. The primary tumor size was 12 × 10 × 8 cm. The patient had received three times gelfoam embolization in July 2011, April 2012, and December 2013, respectively. Three years of follow-up indicated that the development of tumor (13 × 10 × 8 cm) was slow-growing after treatment (Fig. 2).

**Fig. 3:**
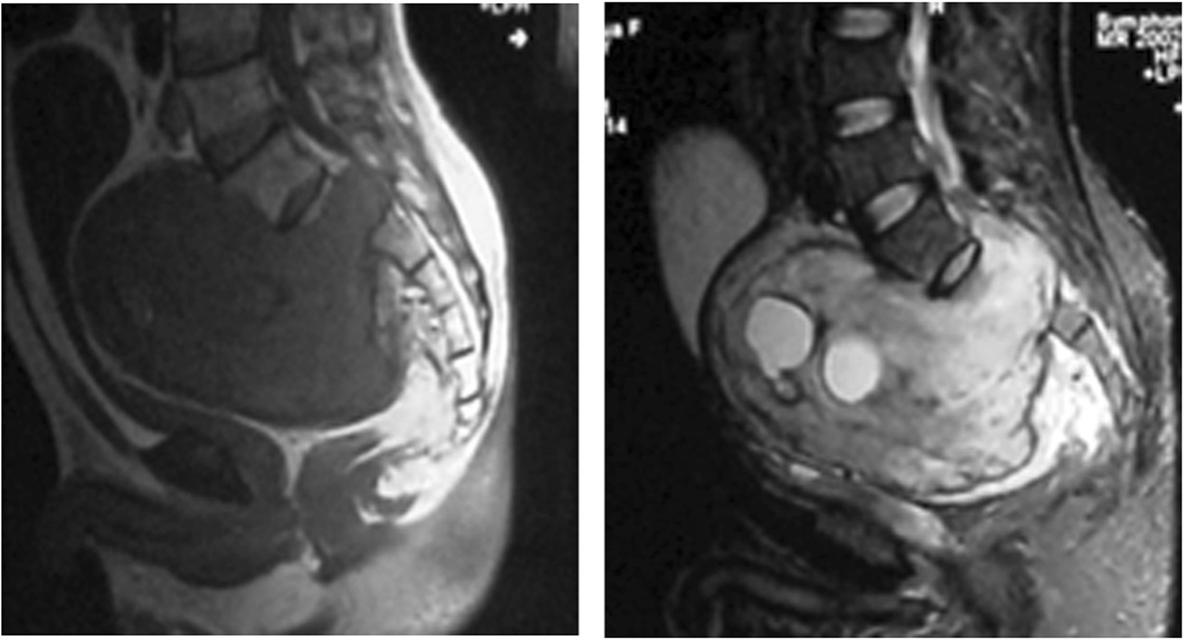
The magnetic resonance imaging (MRI) of patient C after gelatin sponge particle (GSPs) implantation taken in July 2014

Patient C (51 years old, male), whose sacral lump were detected during routine physical examination, was diagnosed as sacral schwannoma with a tumor size of 12 × 9 × 6 cm. Then, the patient had received three treatments of gelfoam embolization in July 2012, April 2013, and January 2014, respectively. With a 2-year follow-up, the tumor (13 × 10 × 6 cm) was slowly aggressive (Fig. 3).

## Discussion

Sacral tumors were rare pathologies, and the management of the tumor generated a complex medical problem, which were usually diagnosed in advanced stages with extended dimensions involving the surrounding organs and sacral nerves [15, 16]. An early diagnosis and treatment were of great help for the survival of patients. However, the treatment of sacral neurogenic tumors was still controversial. Surgical treatment could obtain a complete surgical resection. Nevertheless, this treatment was always restricted due to extensive intraoperative blood loss and serious complications. In the present study, we performed surgical and non-surgical treatments for patients with sacral neurogenic tumor. Our results showed that there was a slow development of tumors for patients who received gelfoam embolization or gelfoam embolization combined with CT-guided ^125^I seed implantation and none of patients suffered a local relapse.

Due to the risks brought by surgical resection, palliative treatment was considered to be an effective treatment for tumors, such as TAE and radioactive ^125^I seed implantation. Butori et al. [17] had reported that preoperative uterine artery embolization could reduce blood loss during surgery and improve the chances of performing conservative surgery. Velmahos et al. [18] had applied angiographic embolization (AE) to the treatment of patients with abdominal visceral organ injuries or major pelvic fractures. Their results indicated that AE, as a reasonable and safety method, was highly effective in preventing bleeding caused by injuries. Besides, gelfoam, a safe and temporary water-insoluble hemostatic agent, was widely used in isolation or as an adjuvant of other permanent agents to obstruct vessels communicated with tumors [19]. Kwon et al. [20] had reported that preoperative GSPs or polyvinyl alcohol particles embolization was effective to reduce intraoperative and postoperative blood loss in patients with hypervascular metastasis to long bones. In our study, patients B and C, who were diagnosed with neurofibroma and schwannoma respectively, both had received three times gelfoam embolization. Later follow-up results demonstrated that the tumors were moving slowly. The tumor sizes were reduced after treatment when compared to the primary size. Thus, our results suggested that gelfoam embolization could achieve a certain degree of efficacy on prevention of sacral tumor growth.

Additionally, it had been reported that CT-guided brachytherapy using ^125^I seed implantation, as a safe, effective, and uncomplicated treatment, could reduce the pain for patients with advanced pancreatic cancer [21]. Zhang et al. [22] had found the combination of ^125^I seed implantation and gemcitabine plus cisplatin chemotherapy was safe and effective for patients with advanced non-small cell lung cancer. Jiang et al. [23] concluded that CT-guided ^125^I seed implantation was a feasible and safe treatment approach for patients with recurrent head and neck cancers. In the present study, one case with giant sacral neurofibroma was treated with CT-guided ^125^I seed implantation and GSP embolization, and the results demonstrated the development of tumors were slow for 5 years following up, which indicated that CT-guided ^125^I seed implantation and GSP embolization were effective treatment strategies.

However, there were some limitations in our study. Firstly, this retrospective study lack of complete data. Secondly, all the patients received conservative treatment were benign tumors, so the effects of CT-guided ^125^I seed implantation or GSPs embolization on malignancy are required to be confirmed in future experiments. Thirdly, due to the lack of patients treated with CT-guided ^125^I seed implantation alone, the differences between ^125^I seed implantation and GSPs embolization for treatment of patients were unknown, which is also needed to be further explored.

## Conclusions

In summary, a retrospective study was performed for patients with giant benign sacral neurogenic tumors to assess the prognosis after gelfoam embolization or gelfoam embolization combined with CT-guided ^125^I seed implantation. The results revealed that non-surgical treatment was an effective method for preventing the progress and development of giant benign sacral neurogenic tumors. Therefore, it is worth thinking twice whether the patient with giant benign sacral neurogenic tumors need to perform a surgery.

### Highlights

1. Nineteen cases received surgical and five cases received non-surgical treatment.
2. Two of the five patients were treated thrice with GSP embolization.
3. GSPs embolization combined with ^125^I seed implantation was performed in one case.
4. Conservative treatment could slow down the development of benign sacral tumors.

## Abbreviations

125I: Iodine-125
CT: computed tomography
GSPs: gelatin sponge particles
KPS: Karnofsky performance status
MRI: magnetic resonance imaging
PSR: partial sacral resection
TAE: transcatheter arterial embolization
